# Supplementary material for: Study on the corrosion behavior and mechanical response of weakly cemented sandstone in alkaline solutions
Source: PLoS One. 2024 Sep 4;19(9):e0309544. doi: 10.1371/journal.pone.0309544 (PMC11373863; doi:10.1371/journal.pone.0309544)
Supplement: S1 File — (DOCX) [file pone.0309544.s001.docx]

Study on the corrosion behavior and mechanical response of weakly cemented sandstone in alkaline solutions

Jie Zhang^1^, Qingsong Zhuo^1,*^, Qian Zheng^2^, Mingang Zhang^3^, Xiaoyu Zhao^3^, Jigang Geng^3^, Xiaoshi Li^3^ , Ruoyu Bao^4^

^1^ College of Energy, Xi’an University of Science and Technology, Xi’an, China

^2^ College of Intelligent Manufacturing and Information Engineering, Shaanxi Energy Institute, Xian Yang, China

^3^ Department of medical imaging, Xi’an Daxing Hospital,Xi’an, China

^4^ Information Institute of the Ministry of Emergency Management of the PRC, Beijing, China

* [zhuo_simon@163.com](mailto:zhuo_simon@163.com)

**Experimental materials, equipment, and methods**

**Rock specimens and experimental equipment**

The weakly cemented sandstone samples required for this experiment were collected from the roof rock layer of the 30208 belt conveyor roadway in Longhua Coal Mine, Sunjiacha Town, Yushenfu mining area in Northern Shaanxi. The cores were taken from the roof according to the distribution and structural characteristics of the coal seams in the mine field, and all selected cores were from the same location within the roadway. After meticulous classification and identification, the cores were processed in the laboratory into standard cylindrical specimens with diameters of 50 mm and heights of 100 mm (for compression), 25 mm (for tension), and 50 mm (for shear) according to the national “Standard for Test Methods of Rock” (GB/T 50218-2014). All specimens were precision machined to ensure that their end face perpendicularity, parallelism, and flatness met the precision required by national standards. After removing any visible defects, the average density of the specimens in their natural water-containing state was 2.66 g/cm³. Mechanical property testing was completed at the MTS laboratory of Xi'an University of Science and Technology, using an HCT series microcomputer controlled electro-hydraulic servo universal testing machine. This machine is equipped with an advanced electro-hydraulic servo control system, which can achieve closed-loop control of load and displacement to ensure high precision of the experiments. During the immersion of rock samples in chemical solutions, the pH was measured using a high-precision pH meter provided by Lichen Technology, with a highly sensitive probe and three-point calibration function ensuring measurement accuracy to 0.01. The mass change of the rock specimens was monitored by a Mengchuang electronic scale with an accuracy of 0.1 g. SEM scanning analysis was conducted using an Axio Scope.A1 polarized light microscope to investigate the microstructural characteristics of weakly cemented sandstone under various alkaline immersion conditions. The operational parameters of the microscope comprised a principal voltage of 100V, power consumption of 140W, with specimen dimensions required to be at least 30mm×60mm×90mm. Additionally , a thin section slicing thickness of 0.03mm was adopted to guarantee clear observation and meticulous analysis of the intricate structures. Concurrently, through the application of pseudocolor enhancement technology, the visual quality of SEM images was improved, enabling a more intuitive analysis and description of the microscale damage and corrosion in the rock mass. Fig 1 displays the processing and testing equipment for the rock specimens.


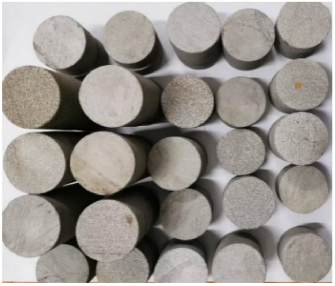

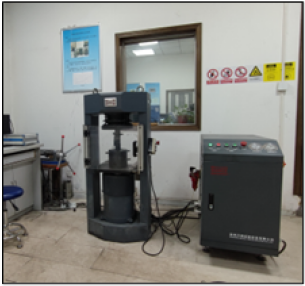

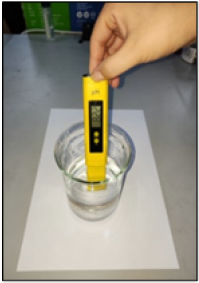

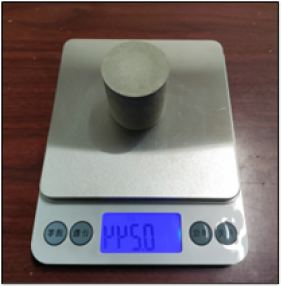

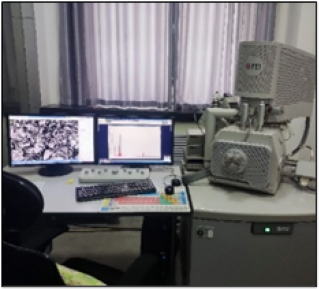


**E**

**A**

**D**

**C**

**B**

**Fig 1. Rock specimens and testing equipment.** (A) Processed specimen. (B) Pressure testing machine. (C) pH testing pen. (D) Electronic scale. (E) Polarized light microscope.

**Preparation of chemical solutions**

To verify the types of groundwater in the mining areas of Northern Shaanxi, this study selected the seepage water from the roof of the 3^-1^ coal seam in Longhua Mine, Shenmu City for chemical composition analysis. Field measurements indicated that the average annual temperature in the 3^-1^ coal seam roadway is approximately 20.8°C, with relative humidity ranging from 93% to 95%. When collecting water samples, 500 mL plastic bottles, rinsed three to four times with the raw water and corresponding samples, were used. The pH value of the water samples was determined using an onsite water quality monitor and preserved under sealed conditions at temperatures between 0 to 4°C. Sample filtration was conducted using a microporous filter membrane with a diameter of 0.45μm for cation testing. The samples were treated with dilute nitric acid in the laboratory, and the concentrations of anions (SO2-4,Cl^-^) were determined by ion chromatography, while the concentration of HCO- 3 was measured by titration; cation concentrations (Ca^2+^, Na^+^, Mg^2+^) were tested using an inductively coupled plasma emission spectrometer. To ensure the accuracy of the tests, an ion conservation analysis was conducted. If the ratio of ion concentration was within a 5% range, it was considered conserved; otherwise, the measurement was retaken. The groundwater exhibited a slightly alkaline nature, with an average pH value of 8.75 for the 3^-1^ coal seam. The ion concentration test results indicated a high degree of mineralization, primarily characterized by high contents of Ca^2+^, Na^+^, Mg^2+^, Cl^-^, SO2- 4, and HCO- 3, with the water chemistry type being predominantly HCO- 3-Ca-Na type.

**Table 1. Chemical characteristics of groundwater in the longhua mine's 3^-1^ coal seam.**

| **pH value and ion concentration** | | **3^-1^ coal seam** |
| --- | --- | --- |
| pH | | 8.75 |
| K^+^ | mg/l | 61.01 |
| Na^+^ | mg/l | 416.92 |
| Ca^2+^ | mg/l | 101.18 |
| Mg^2+^ | mg/l | 93.79 |
| SO2- 4 | mg/l | 590.64 |
| Cl^-^ | mg/l | 1728 |
| HCO- 3 | mg/l | 673.39 |
| NO- 3 | mg/l | 66.04 |
| NO- 2 | mg/l | 20.21 |

To simulate the impact of the alkaline hydrochemical environment of shallow coal seams on the mechanical properties of surrounding rocks, solutions with varying ion concentrations and pH levels were prepared, taking into consideration the primary cations (K⁺, Na⁺, Ca²⁺, Mg²⁺) and anions (Cl^-^, SO2- 4, HCO- 3) present in the percolating water of the 3^-1^ coal seam roof. Solutions with pH values of 7, 9, and 11 were prepared by adding corresponding salts, including anhydrous calcium chloride, sodium chloride, magnesium chloride, and potassium sulfate. These solutions were used to immerse the surrounding rock samples, aiming to observe changes in rock mechanical properties under different alkaline conditions. Deionized distilled water was prepared using the advanced ultrapure water production system at Xi'an University of Science and Technology's laboratory. The equipment used for immersing the samples was a DZF-1 vacuum drying oven. Fig 2 illustrates the drying process of the immersed samples.

**Table 2. Preparation scheme of water chemical solutions.**

| **Solution type** | **Solution components** | **Solution pH value** | **Solution concentration (mg/l)** |
| --- | --- | --- | --- |
| Hydrogen ion concentration of 7 solution | K_2_SO_4_、CaCl_2_、NaCl、MgCl_2_ | 7 | 0.1 |
| Hydrogen ion concentration of 9 solution | K_2_SO_4_、CaCl_2_、NaCl、MgCl_2_ | 9 | 0.1 |
| Hydrogen ion concentration of 11 solution | K_2_SO_4_、CaCl_2_、NaCl、MgCl_2_ | 11 | 0.1 |


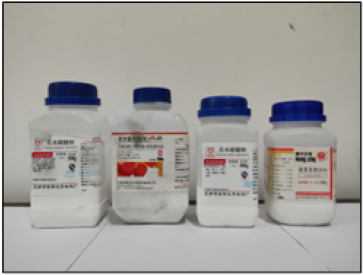

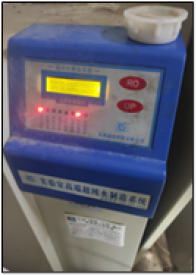

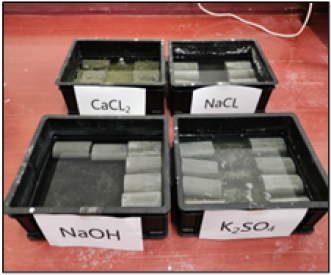

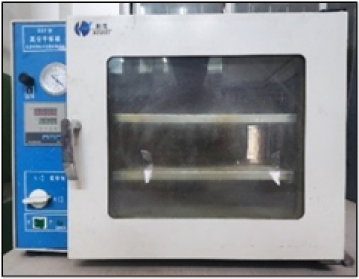


**C**

**D**

**A**

**B**

**Fig 2. Soaking materials and drying equipment.** (A) Ion concentration preparation chemicals. (B) Ultrapure water manufacturing equipment. (C) Soaked rock specimens. (D) DZF vacuum drying oven.

**Experimental method**

Regarding the experimental procedure, rock samples were placed in open PPS acid-base immersion tanks to ensure continuous contact between the solution and the external environment. Air conditioning was employed to control the indoor temperature. During the immersion process, regular records of the mass variation (*Δm*) of the samples were maintained, and data changes were monitored. The pH value of the immersion solution was measured using a pH meter, and the trend of pH changes was recorded. After immersion, the rock samples underwent drying and polishing treatments to ensure that the end faces and side surfaces met testing standards and maintained a smooth and vertical appearance.Prior to conducting uniaxial compression tests on the rock samples, a uniform application of Vaseline was applied to both the upper and lower ends of the samples to minimize friction's influence on the test results. The testing employed a displacement loading method, with the upper loading device kept stable, and a lower loading rate of 0.06 millimeters per minute was applied. Throughout this process, the axial load and axial displacement of the rock sample were monitored in real-time, and deformation and failure processes were recorded. Data changes and numerical variations were also concurrently documented and analyzed.
