# Supplementary material for: Study on the corrosion behavior and mechanical response of weakly cemented sandstone in alkaline solutions
Source: PLoS One. 2024 Sep 4;19(9):e0309544. doi: 10.1371/journal.pone.0309544 (PMC11373863; doi:10.1371/journal.pone.0309544)
Supplement: Attachment — Submitted filename: PONE-D-24-04355-R2.pdf [file pone.0309544.s004.pdf]

Review of MS #PONE-D-24-04355_R1

Study on the corrosion behavior and mechanical response of weakly cemented sandstone in alkaline solutions

By Qingsong Zhuo et al.

Recommendation: Publish with revisions suggested below.

Comments

Some of my concerns have been addressed and satisfied. Below, I summaries my remaining concerns in red:

- "The authors focus mostly on recording the mass variation (Δm) in the rock samples over a long period of time -- 55 days, e.g. figure 3. However, it is also customary to present and analyze correlations as a function of porosity, see *Qi Ping et al. Applied Science 2022, 12, 7635; Yun Lin et al. Hindawi Geofluids 2019, 7320536.* Although the authors mention the pdf of porosity, e.g. Table 4, they do not present any correlations against porosity or discussion."

  Although the authors have updated the review section, what I really wanted was actual plots of variable against the porosity such as those appearing in the cited references. Please address this point with more analysis and correlations against porosity.

- Figure 3: (D) is simply a repeat of A-C with fits added. Please reduce this to a single figure3D=figure 3.

  Figure 3: make the axes and table insert fonts bigger (or put the table into the main text) – they are not clearly visible.

  All figures: increase the axes font size for the same reason as above.

- "About 90% of the references are studies in China published in Chinese journals; I have found these difficult to access, many I could not access at all. It is important that references, particularly those containing data, in an internationally renowned journal such as PONE should be readily accessible to the international community. The authors should make sure that their references are accessible internationally."

  The two references that I suggested were just examples. The authors should find more similar references.

  Although the authors have referenced more international journals, there are still some that are difficult to access e.g. references 16. Furthermore, some of the web links are spurious – for example, the links in references 38-42 seem to be wrong. I do not have the time to go through all the references – the authors should make sure that all 42 references are readily accessible internationally and are properly linked.
