# Supplementary material for: Study on the corrosion behavior and mechanical response of weakly cemented sandstone in alkaline solutions
Source: PLoS One. 2024 Sep 4;19(9):e0309544. doi: 10.1371/journal.pone.0309544 (PMC11373863; doi:10.1371/journal.pone.0309544)
Supplement: Attachment — Submitted filename: PONE-D-24-04355-Review.docx [file pone.0309544.s002.docx]

**Review of MS #PONE-D-24-04355**

**Study on the corrosion behavior and mechanical response of weakly cemented sandstone in alkaline solutions**

**By Qingsong Zhuo et al.**

**Reviewer Recommendation:** Reject.

**Summary of paper**

This is an experimental study to assess the impact of varying alkaline pH levels on macroscopic mass damage, microscopic pore characteristics, and uniaxial compressive strength of weakly cemented sandstone. This work fills a gap in studies on weakly cemented sandstone in the Shaanbei mining area.

The results indicate that as the alkalinity of the solution increases, the mass damage to the rock intensifies. However, in the pH range of 9 to 11, the mass loss rate is mitigated due to the clogging of pores by complexes formed by cations such as Ca2+ and Mg2+. Microstructural analysis reveals that porosity, pore size, and roundness undergo changes with increasing alkalinity; native pores increase, secondary pores develop, and the roundness of the pores shows a declining trend, with pore shapes evolving from elongated to elliptical and even spherical. Moreover, both the uniaxial compressive strength and elastic modulus of the rock exhibit a downward trend with increasing pH values.

**Comments**

- The topic is within the scope of PONE.
- The paper is generally well written, the level of English is good.
- The experimental facilities and methods are well described.
- The authors propose various chemical pathways that could explain the results.
- The authors focus mostly on recording the mass variation ($\Delta m$) in the rock samples over a long period of time -- 55 days, e.g. figure 3. However, it is also customary to present and analyze correlations as a function of porosity, see *Qi Ping et al. Applied Science 2022, 12, 7635; Yun Lin et al. Hindawi Geofluids 2019, 7320536.* Although the authors mention the pdf of porosity, e.g. Table 4, they do not present any correlations against porosity or discussion.
- Why did the authors not consider the effects of acidic solutions? That would complete the whole range of pH, and presumably relatively straight forward given that they already have the setup.
- About 90% of the references are studies in China published in Chinese journals; I have found these difficult to access, many I could not access at all. It is important that references, particularly those containing data, in an internationally renowned journal such as PONE should be readily accessible to the international community. The authors should make sure that their references are accessible internationally.
- Along the same lines as above, references to other comparable rock structures in other parts of the world should also be made compared and conclusions drawn. How do their findings compare with other similar rocks from around the world? Are the results predictable, or is there anything radically different or surprising?

**Summary and recommendation**

This paper is essentially about characterizing the development of corrosion and failure in weakly cemented sandstone form the Shaanbei mining area. Unfortunately, many deficiencies in the paper compel me to recommend rejection for publication in PONE:

(1) A comparison with similar studies worldwide is lacking.
(2) A discussion of porosity with plots/figures of correlations against porosity is lacking.
(3) References and comparison with similar rock structures from other parts of the world is lacking.
(4) References to readily accessible internationally peer reviewed journals must be made.
