# Supplementary material for: Study on the corrosion behavior and mechanical response of weakly cemented sandstone in alkaline solutions
Source: PLoS One. 2024 Sep 4;19(9):e0309544. doi: 10.1371/journal.pone.0309544 (PMC11373863; doi:10.1371/journal.pone.0309544)
Supplement: Attachment — Submitted filename: Response to Reviewers.docx [file pone.0309544.s003.docx]

Dear editors and reviewers:

I am delighted to receive your response and am deeply appreciative of the effort and diligent approach you have demonstrated in reviewing my article. Moving forward, I intend to address each question raised by the reviewing experts systematically. In accordance with your suggestions, I have meticulously revised the manuscript, and herein, I provide responses to your comments for your review and verification.

**Response to Reviewer 1 Comments**

**Point 1:** Please check the logic of the abstract. It should clearly describe the research background, the method used to conduct the research, the main content of the manuscript, the results, and the expected effect. The current version should be carefully simplified.

**Response 1:** I deeply appreciate your constructive feedback on the abstract. In line with your suggestion, I have meticulously reviewed and revised the abstract to clearly lay out the research background, explicitly detail the methodologies used, accurately summarize the manuscript's core contents, plainly present the attained results, and succinctly communicate the anticipated outcomes.

**Point 2:** The introduction listed many references that are mainly related to the corrosion behavior and mechanical response of weakly cemented sandstone in alkaline solutions. If the authors would like to keep these references, some discussions on the relevance of these refs to the present research are needed. A review of the directly relevant refs will be more helpful for the reader.

**Response 2:** Thank you for highlighting the need for a more nuanced discussion of the references cited in the introduction. I understand the importance of demonstrating the relevance of each reference to our present work. Consequently, I have critically reviewed the list and augmented the introductory section with a concise discourse on how each of the selected references connects to our study objectives, methodologies, and anticipated findings. This additional context should enhance readability and clarify the rationale behind the inclusion of these references, thereby offering readers a more coherent understanding of the research landscape within which our work is situated.

**Point 3:** The scanning electron microscope (SEM) analysis method utilized in this paper is particularly effective for corrosive environments. On the other hand, this SEM analysis technique is well-established. However, the study does not provide details such as the equipment model and other parameters. The current research represents a direct application of this method, yet it fails to adequately describe the microscopic morphology and structure of the rock texture. The existing descriptions are solely based on the structure of the magnified rock texture, without a specific quantification process.

**Response 3:** I appreciate the reviewer's comment concerning the SEM analysis method. In addressing this, I have included detailed specifics about the SEM equipment model, operational voltage levels, and magnification configurations employed in my experiments within the Materials and Methods section. Furthermore, I have enhanced the depiction of the rock texture's microscopic morphology and structure by integrating quantitative measures, such as analyses of pore size distribution and surface roughness, facilitated by image processing techniques. These improvements are aimed at furnishing a more holistic and scientifically rigorous portrayal of the SEM analysis outcomes.

**Point 4:** In the analysis of failure patterns in uniaxial compression tests on weakly cemented sandstone, the characterization of the stress-induced failure modes for rock samples needs enhancement. The details within this section should be further elaborated upon.

**Response 4:** I am genuinely appreciative of the reviewer's astute recommendation emphasizing the need for a more exhaustive depiction of stress-induced failure mechanisms in my uniaxial compression experiments concerning weakly cemented sandstone samples. In light of this guidance, I have comprehensively enhanced the explication of the complex breakdown processes triggered by loading in the course of conducting uniaxial compression tests on these weakly consolidated sandstone specimens.

**Point 5:** It is noted that your manuscript needs careful editing, particularly attention to English grammar, spelling, and sentence structure so that the goals and results of the study are clear to the reader. It is strongly recommended that simple and precise sentences be used to convey the study's findings.

**Response 5:** I am deeply grateful for the reviewer's meticulous scrutiny of linguistic clarity and the constructive criticism offered. Special attention has been devoted to ensuring grammatical precision, rectifying spelling mistakes, and simplifying sentence structures for improved readability. Consequently, I have reformulated several sections, adopting simpler and more succinct sentences, with the aim of conveying our study's aims, methodology, results, and implications more lucidly. I am assured that these modifications will markedly enhance the manuscript's clarity and efficacy in communicating our research to the targeted readership.

**Response to Reviewer 2 Comments**

**Point 1:** The authors focus mostly on recording the mass variation () in the rock samples over a long period of time -- 55 days, e.g. figure 3. However, it is also customary to present and analyze correlations as a function of porosity, see Qi Ping et al. Applied Science 2022, 12, 7635; Yun Lin et al. Hindawi Geofluids 2019, 7320536. Although the authors mention the pdf of porosity, e.g. Table 4, they do not present any correlations against porosity or discussion.

**Response 1:** I am deeply grateful to the reviewer for suggesting a more in-depth analysis regarding the correlation with porosity. In response to this feedback, I have conducted additional statistical evaluations, referencing and citing the similar approaches employed by Qi Ping et al. (Applied Science, 2022, 12, 7635) and Yun Lin et al. (Hindawi Geofluids, 2019, 7320536). This detailed analysis encompasses considerations such as the transformation of pore shape factors, effects of mineral dissolution and precipitation, thereby enriching the discussion on this topic.

**Point 2:** Why did the authors not consider the effects of acidic solutions? That would complete the whole range of pH, and presumably relatively straight forward given that they already have the setup.

**Response 2:** The reviewer poses a relevant question about the omission of acidic solutions in my study. My initial emphasis rested on elucidating corrosion characteristics in alkaline environments, given their predominance in the targeted geological context. Nonetheless, I acknowledge the importance of broadening my inquiry to cover the complete pH spectrum. In light of this realization, I intend to conduct further experiments incorporating acidic solutions in a subsequent study. These additional investigations will serve to augment the present results and facilitate a holistic comprehension of the rock's behavior throughout the entire pH range. I am thankful for the reviewer's insight, which has significantly expanded the horizon of my impending research efforts.

**Point 3:** About 90% of the references are studies in China published in Chinese journals; I have found these difficult to access, many I could not access at all. It is important that references, particularly those containing data, in an internationally renowned journal such as PONE should be readily accessible to the international community. The authors should make sure that their references are accessible internationally.

**Response 3:** I am exceedingly grateful for your insightful comments on the accessibility of references in my paper, particularly highlighting the challenges that referencing Chinese journals might impose on an international audience. Your observation has both inspired me and proved vital in informing necessary enhancements to my work.

In line with your guidance, I have taken the following actions: I have meticulously verified and corrected the URLs for the Chinese references to ensure seamless linking and accessibility. Furthermore, I have expanded the bibliography to incorporate a greater number of foreign language references relevant to the study, thereby elevating the paper's international scope and perspective.

**Point 4:** Along the same lines as above, references to other comparable rock structures in other parts of the world should also be made compared and conclusions drawn. How do their findings compare with other similar rocks from around the world? Are the results predictable, or is there anything radically different or surprising?

**Response 4:** Sincere gratitude for emphasizing the importance of positioning my research findings within a global context. Heeding your invaluable advice, I have integrated the following enhancements into the manuscript, thereby augmenting its depth and breadth through the inclusion of a comparative analysis with analogous geological structures worldwide.I have broadened the scope of the literature review to encompass pivotal global studies that concentrate on geological formations resembling my rock layers. These studies meticulously compare and contrast similarities and differences in geological attributes, formation processes, and observable behaviors.

I am immensely grateful to the editors and reviewers for their invaluable advice, which has enabled me to meticulously revise the manuscript, restructure its content, and enhance both the academic rigor and readability of the paper. I extend my sincere thanks once again to the editorial team and reviewers for their diligent efforts. Each of your comments has proven to be immensely valuable and instrumental in refining and improving our work. I remain hopeful that our paper will soon gain full recognition and be published.

Dr. Qingsong Zhuo

Xi'an City, Shaanxi Province, China

Jul. 2, 2024
