# Supplementary material for: Study on the corrosion behavior and mechanical response of weakly cemented sandstone in alkaline solutions
Source: PLoS One. 2024 Sep 4;19(9):e0309544. doi: 10.1371/journal.pone.0309544 (PMC11373863; doi:10.1371/journal.pone.0309544)
Supplement: Attachment — Submitted filename: Response to Reviewers.docx [file pone.0309544.s005.docx]

Dear editors and reviewers:

I am delighted to receive your response and am deeply appreciative of the effort and diligent approach you have demonstrated in reviewing my article. Moving forward, I intend to address each question raised by the reviewing experts systematically. In accordance with your suggestions, I have meticulously revised the manuscript, and herein, I provide responses to your comments for your review and verification.

**Response to Reviewer Comments**

**Point 1:** “The authors focus mostly on recording the mass variation (Δm) in the rock samples over a long period of time -- 55 days, e.g. figure 3. However, it is also customary to present and analyze correlations as a function of porosity, see Qi Ping et al. Applied Science 2022, 12, 7635; Yun Lin et al. Hindawi Geofluids 2019, 7320536. Although the authors mention the pdf of porosity, e.g. Table 4, they do not present any correlations against porosity or discussion.”

Although the authors have updated the review section, what I really wanted was actual plots of variable against the porosity such as those appearing in the cited references. Please address this point with more analysis and correlations against porosity.

**Response 1:** Thank you for your valuable feedback on our manuscript. We have addressed your comments as follows:We recognize the importance of presenting and analyzing correlations with porosity. We have provided detailed scatter plots illustrating the relationship between porosity and various parameters, such as pore diameter, pore area, and circularity. These plots were inspired by the methodology employed in the references you cited (Qi Ping et al., Applied Science 2022, 12, 7635; Yun Lin et al., Hindawi Geofluids 2019, 7320536). We have expanded the discussion section to include a comprehensive analysis of these correlations. We have provided interpretations for the observed trends and their implications for the structural changes in the rock samples under different immersion conditions. This detailed discussion, as you requested, addresses the need for a more thorough analysis and correlation with porosity.

We are grateful for your valuable feedback. We look forward to your further comments and suggestions.

**Point 2:** Figure 3: (D) is simply a repeat of A-C with fits added. Please reduce this to a single figure3D=figure 3.Figure 3: make the axes and table insert fonts bigger (or put the table into the main text) – they are not clearly visible. All figures: increase the axes font size for the same reason as above.

**Response 2:** Thank you for your suggestion. I have enlarged and standardized the font size in all figures throughout the paper to ensure clarity and consistency for reader accessibility.

**Point 3:** “About 90% of the references are studies in China published in Chinese journals; I have found these difficult to access, many I could not access at all. It is important that references, particularly those containing data, in an internationally renowned journal such as PONE should be readily accessible to the international community. The authors should make sure that their references are accessible internationally.”

The two references that I suggested were just examples. The authors should find more similar references. Although the authors have referenced more international journals, there are still some that are difficult to access e.g. references 16. Furthermore, some of the web links are spurious – for example, the links in references 38-42 seem to be wrong. I do not have the time to go through all the references – the authors should make sure that all 42 references are readily accessible internationally and are properly linked.

**Response 3:** I have carefully checked and revised all references to guarantee that they are correctly formatted, hyperlinked, and lead to the correct online sources.

I am immensely grateful to the editors and reviewers for their invaluable advice, which has enabled me to meticulously revise the manuscript, restructure its content, and enhance both the academic rigor and readability of the paper. I extend my sincere thanks once again to the editorial team and reviewers for their diligent efforts. Each of your comments has proven to be immensely valuable and instrumental in refining and improving our work. I remain hopeful that our paper will soon gain full recognition and be published.

Dr. Qingsong Zhuo

Xi'an City, Shaanxi Province, China

Aug. 5, 2024
